# Supplementary material for: Patients’ and Relatives’ Preferences for a Palliative/Oncology Day Ward and Out-of-Hours Telemedicine—An Interpretive Description
Source: Healthcare (Basel). 2021 Jun 18;9(6):758. doi: 10.3390/healthcare9060758 (PMC8235271; doi:10.3390/healthcare9060758)
Supplement: Supplementary file 1 [file healthcare-09-00758-s001.zip › healthcare-1208399-supplementary.pdf]

Supplementary material:

Table S1. Interview guide.

Area	Question	Prompts –if necessary
Question 1 – day ward	What do you think about receiving treatment on a day ward rather than being admitted to the ward for treatment?	
Question 2 - telemedicine	What do you think about using a screen* to contact staff on the ward when the day ward is closed?	
Question 3 - Arrival	What are your preferences in relation to the lay out of the day ward?	- Signs, waiting room<br>- Opening hours<br>- Reception<br>- Information on arrival etc<br>- Information to family
Question 4 - Under visit	What are your preferences in during your visit to the day ward?	- Expectations to staff<br>- Information prior to visit<br>- Understandable information<br>- Participation in decision making to the extent that one can<br>- Consent to inform relatives<br>- Managing one's illness
Question 5 – Returning home.	What would be important for you when returning home from a visit to the day ward?	- Information about symptoms, side effects of medicine, treatment plan and follow-up<br>- Consideration of individual needs<br>- Information on how to contact staff<br>- Introduction to telemedicine<br>- Co-operation with GP/district nurse
Closing question	Have you any other comments about your preferences in relation to treatment on a day ward and out-of-hours video consultation support?	
